# A framework and model system to investigate linear system behavior in *Escherichia coli*

**DOI:** 10.1186/1754-1611-5-3

**Published:** 2011-04-22

**Authors:** Meghdad Hajimorad, Paul R Gray, Jay D Keasling

**Affiliations:** 1 Department of Electrical Engineering and Computer Sciences, University of California, Berkeley, CA 94720, USA; 2 Synthetic Biology Engineering Research Center, University of California, Berkeley, CA 94720, USA; 3 QB3: California Institute for Quantitative Biological Research, University of California, Berkeley, CA 94720, USA; 4 Physical Biosciences Division, Lawrence Berkeley National Laboratory, Berkeley, CA 94720, USA; 5 Department of Bioengineering, University of California, Berkeley, CA 94720, USA; 6 Department of Chemical and Biomolecular Engineering, University of California, Berkeley, CA 94720, USA; 7 Joint BioEnergy Institute, Emeryville, CA 94608, USA

## Abstract

**Background:**

The ability to compose biological systems from smaller elements that act independently of the other upon assembly may help make the forward engineering of biological systems practical. Engineering biology in this manner is made difficult by the inherent nonlinear response of organisms to genetic devices. Devices are inevitably coupled to one another in the cell because they share the same transcriptional machinery for expression. Thus, new properties can emerge when devices that had been characterized in isolation are expressed concurrently. We show in this report that, similar to physical systems, the *Escherichia coli *(*E. coli*) transcriptional system can exhibit linear behavior under "small" perturbation conditions. This, in turn, allows devices to be treated as independent modules.

**Results:**

We developed a framework and model system consisting of three devices to investigate linear system behavior in *E. coli*. Our framework employed the transfer curve concept to determine the amount of nonlinearity elicited by the *E. coli *transcriptional system in response to the devices. To this effect, the model system was quantitatively characterized using real-time quantitative PCR to produce device transfer curves (DTCs). Two of the devices encoded the bacterial neomycin phosphotransferase II (*nptII*) and chloramphenicol acetyl transferase (*cat*), while the third encoded the jellyfish-originating green fluorescent protein (*gfp*). The *gfp *device was the most nonlinear in our system, with *nptII *and *cat *devices eliciting linear responses. Superposition experiments verified these findings, with independence among the three devices having been lost when *gfp *was present at copy numbers above the lowest one used.

**Conclusions:**

We show that linear system behavior is possible in *E. coli*. Elucidation of the mechanism underlying the nonlinearity observed in *gfp *may lead to design rules that ensure linear system behavior, enabling the accurate prediction of the quantitative behavior of a system assembled from individually characterized devices. Our work suggests that biological systems follow principles similar to physical ones, and that concepts borrowed from the latter (such as DTCs) may be of use in the characterization and design of biological systems.

## Background

Engineering biological systems with predictable, quantitative behavior is currently a challenging problem. Presently, this requires months (at times years) of trial-and-error type of experiments, with the engineering of functional systems being more akin to art than engineering [1]. Synthetic biology aims to develop foundational principles and technologies that will enable the systematic forward engineering of biological systems [2-4]. In particular, synthetic biology aims to develop frameworks that apply the engineering principles of abstraction, modularity, and composition to biological engineering. Basic abstraction and physical composition frameworks have been applied to the engineering of biology through the use of BioBricks and the Registry of Standard Biological Parts [5-7]. A characteristic feature of other established engineering disciplines is the ability to design and construct systems by way of modularity. The concept of modularity allows engineers to design and build physical systems by bringing together modules that contribute independently to the whole, thereby giving rise to a system whose quantitative behavior can be predicted from its constituent modules [8-10]. A pressing research question is whether the complexity of living organisms allows engineers to design and construct biological systems from smaller elements characterized in isolation [11,12]. The success of synthetic biology as an engineering discipline will depend, in part, on establishing the conditions necessary for this independence property to hold true in living systems [13]. The research contribution of this study is the application of engineering principles towards realizing modularity and functional composition in biological systems. More specifically, we show that genetic devices (each consisting of a promoter, ribosome binding site, gene of interest, and transcription terminator) can behave in a standardized, quantitatively predictable manner. The ability to view devices as modules may be of benefit in such applications as metabolic pathway construction for the production of natural products and other chemicals (microbial chemical factories).

**Figure 1:**
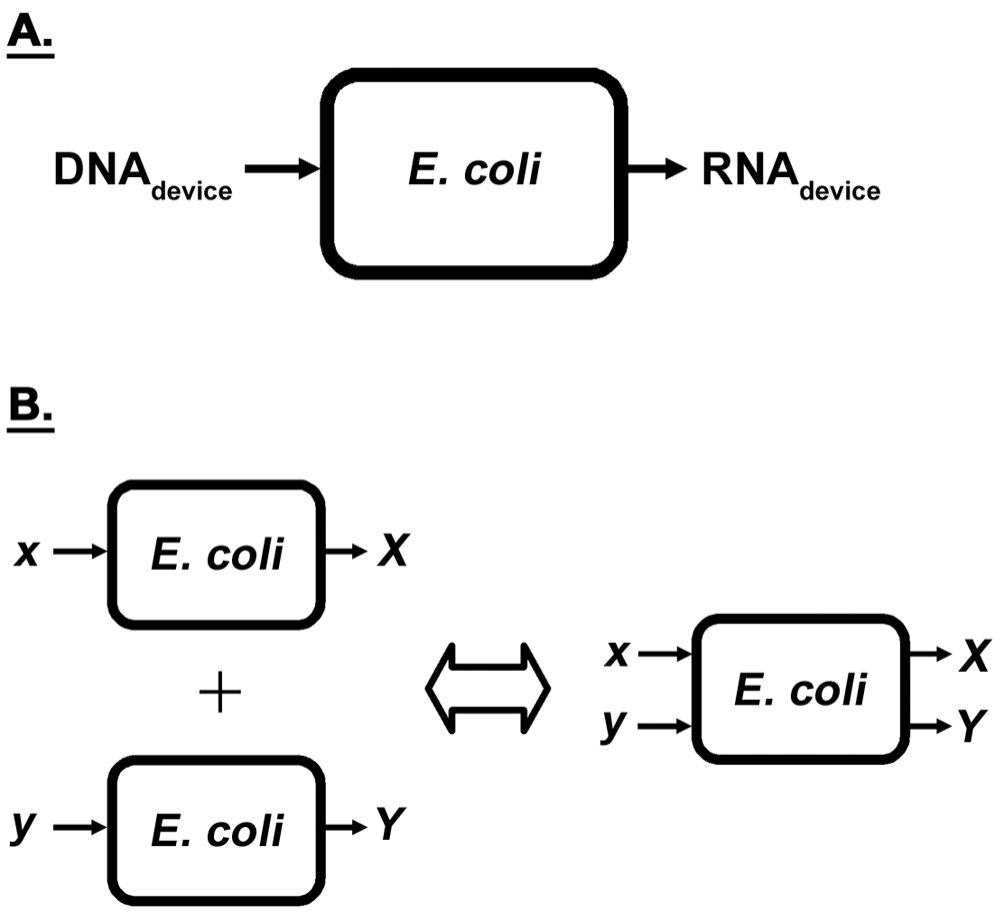
**Black box representation of the *E. coli *transcriptional system**. **A**. Genetic devices (synthetic and native) in the form of DNA act as inputs to the system, with the resultant RNA produced the output. **B**. Pictorial depiction of the superposition principle in effect in linear systems. Superposition states that the output from a set of inputs represents the linear sum of the outputs from each individual input.

Once introduced into the host cell as DNA, a synthetic device must first be expressed by the native transcriptional machinery in order to give rise to the desired function (e.g. production of transcript, desired protein, or metabolites, transduction of a signal, etc.). Synthetic devices introduced into *Escherichia coli *(*E. coli*) for engineering purposes are, in essence, additional devices imposed on top of those present in the wildtype (baseline) case. Synthetic devices can, thus, only begin to behave independently if their respective transcript levels are not affected by the addition of other synthetic devices. This is because of transcription being the initial process in gene expression. In order to motivate the experimental approach taken, the transcriptional machinery of the *E. coli *host cell was viewed as a system in this study. Devices in the form of DNA are its input, with the resultant RNA produced its output (Figure 1A). Synthetic devices, however, are not the only inputs to the system. Thousands of devices are encoded on the *E. coli *genome [14], whose regulated expression allow the organism to survive and grow in a given environment. The same molecular players and building blocks involved in synthesizing RNA encoded by the host's native (chromosomally-encoded) devices are involved in the transcription of synthetic (heterologous) devices. The system (Figure 1A), thus, not only has synthetic devices as inputs, but also the native devices. For independence among devices (synthetic and native) to be possible, the system must be linear, thereby exhibiting the superposition principle by definition [15]. The superposition property of linear systems states that the net response caused by two or more inputs is the sum of the responses that would have been caused by each stimulus individually. That is, if *x *and *y *amounts of DNA for a couple of devices alone produce *X *and *Y *amounts of transcript, respectively, then the concurrent addition of both devices to the system should lead to the formation of *X *and *Y *amounts of transcript (Figure 1B). Systems in practice, however, are nonlinear and do not abide by the superposition principle. As such, the different synthetic and native inputs to the system cannot be studied in isolation. Design for predictable, quantitative behavior would, thus, not only require an understanding of how synthetic devices couple to one another by way of the nonlinearity present in the *E. coli *system, but also how they couple to the host's native devices. Our current level of understanding of these interactions is limited at best qualitatively, much less so in a quantitative manner. This, in part, may explain the difficulty associated with engineering biological systems with predictable, quantitative behavior. As they are embedded inside complex host cells, many interactions are possible between the host cell and introduced constructs.

This study began with the hypothesis that the introduced synthetic devices can be viewed as perturbations to the *E. coli *system. That is, the amount of DNA acting as input to the transcriptional machinery of the host increases with their addition. So long as this increase (i.e., perturbation) is kept "small," the *E. coli *system may perhaps be approximated as a linear one with respect to the introduced synthetic devices, thereby enabling superposition and the decoupling of synthetic devices from one another. This is the small-signal approximation used in the field of electronic circuit design [16]. There, it is used in the design of analog amplifiers, where voltage and current signals act as inputs to nonlinear, transistor-based systems. It should be noted that the copy number of synthetic devices may not be the only factor that perturbs microbial organisms. Promoter strength, ribosome binding site (RBS) strength, gene length, codon usage, and product function are perhaps important factors too. As the intent of our study was to investigate whether *E. coli *can accommodate linear system behavior with standard elements used to genetically modify the organism, we focused on copy number here. Our approach to investigate system nonlinearity involved varying the copy number of devices to generate transfer curves. Nonlinearity of physical systems is often investigated by using transfer curves, where the transfer curve of a system specifies how its output varies with respect to its input under steady-state conditions [16,17]. We show with our approach that concepts applicable to physical systems also apply to biological ones, and that superposition is possible in *E. coli *under certain conditions.

## Results

**Figure 2:**
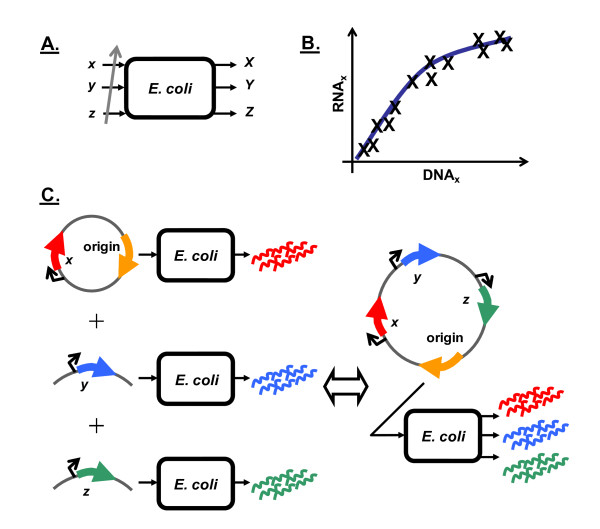
**Experimental approach taken in this study**. **A**. Copy numbers of the genetic devices in the model system were varied and the RNA from a particular device was measured as an output. **B**. DTC for a device (device *x *as an example) was generated by first plotting the RNA produced at each copy number. A regression that minimized the sum of squared residual error was subsequently fitted to the cumulative data. **C**. Linear system behavior was gauged by testing for superposition. Superposition was determined to exist if a correspondence was observed between the left and right hand sides of the depicted figure.

A model system was constructed to enable the introduction of genetic devices into *E. coli *at different copy numbers (Figure 2A). The resultant RNA produced was quantified at each copy number to produce RNA versus DNA device transfer curves (DTCs) (Figure 2B). DTCs were subsequently analyzed to gauge the nonlinearity of the *E. coli *system response to individual devices, with linear system behavior being verified by showing the presence of superposition (Figure 2C).

### Copy number of genetic device varied with plasmid origin of replication

**Figure 3:**
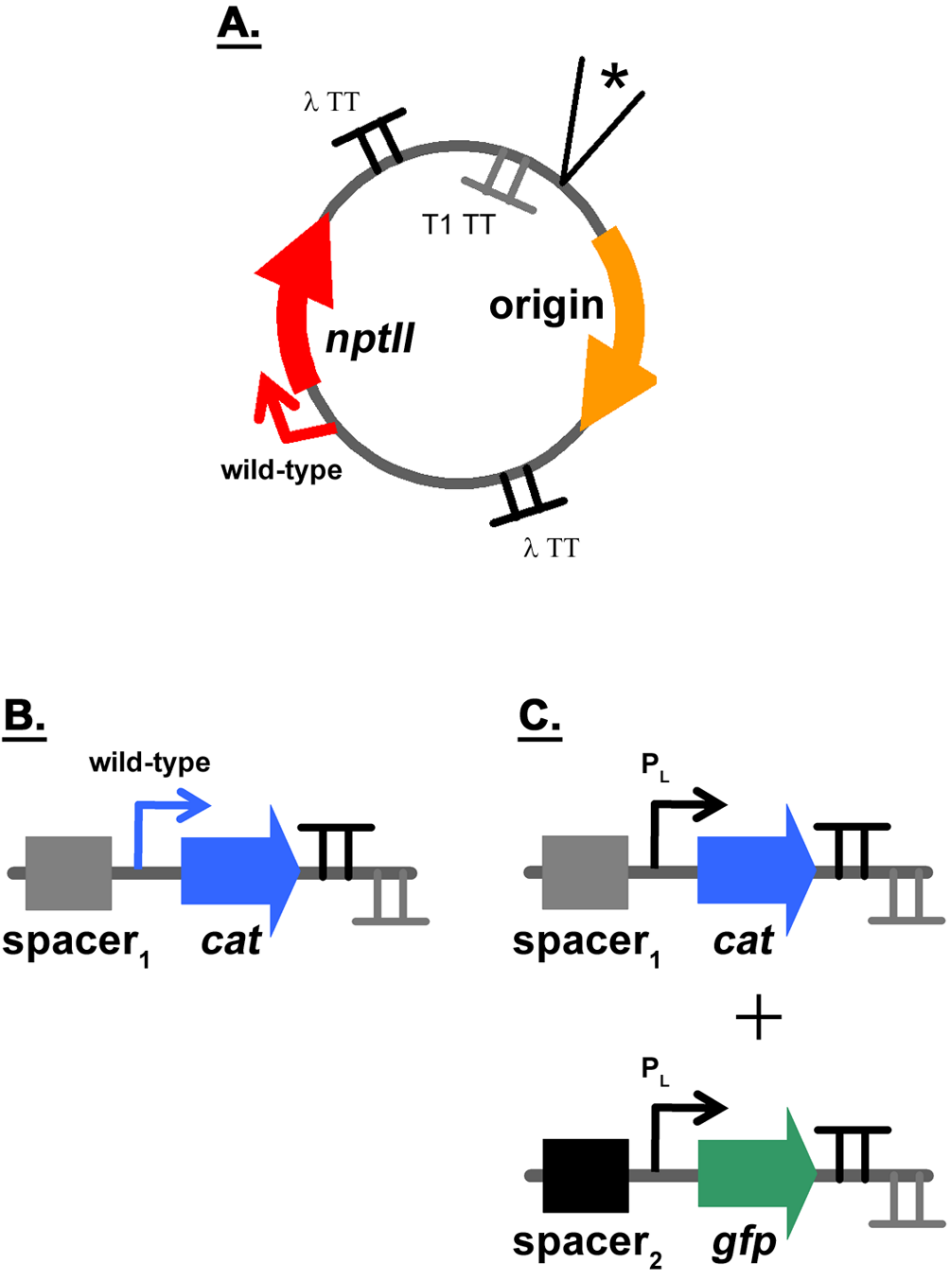
**Diagrams of the plasmids constructed to enable variation of genetic device copy number and to determine DTCs**. **A**. Plasmid backbone with the *nptII *selection marker. The origins of the plasmids pSC101, p15A, pMB1, and pUC were cloned into the backbone using the two unique restriction sites *SmaI *and *AvrII *(not shown). TT denotes transcriptional terminator. Two different terminators (λ t0 and *rrnB *T1) had to be used because cloning attempts aimed at having the same terminator present simultaneously in opposing directions met with failure. The cassettes depicted in panels B and C were cloned into the multi-cloning site indicated by an asterisk. **B**. The *cat *device containing cassette cloned into the backbone. **C**. The *cat *and *gfp *device containing cassettes cloned into the backbone to arrive at the model system. Spacers were used to create spatial separation between neighboring devices (see Methods) to minimize the potential of spatial coupling. The promoter driving *cat *in B is different from that in C. While the native promoter was used in the former, P_L _was used in the latter.

**Figure 4:**
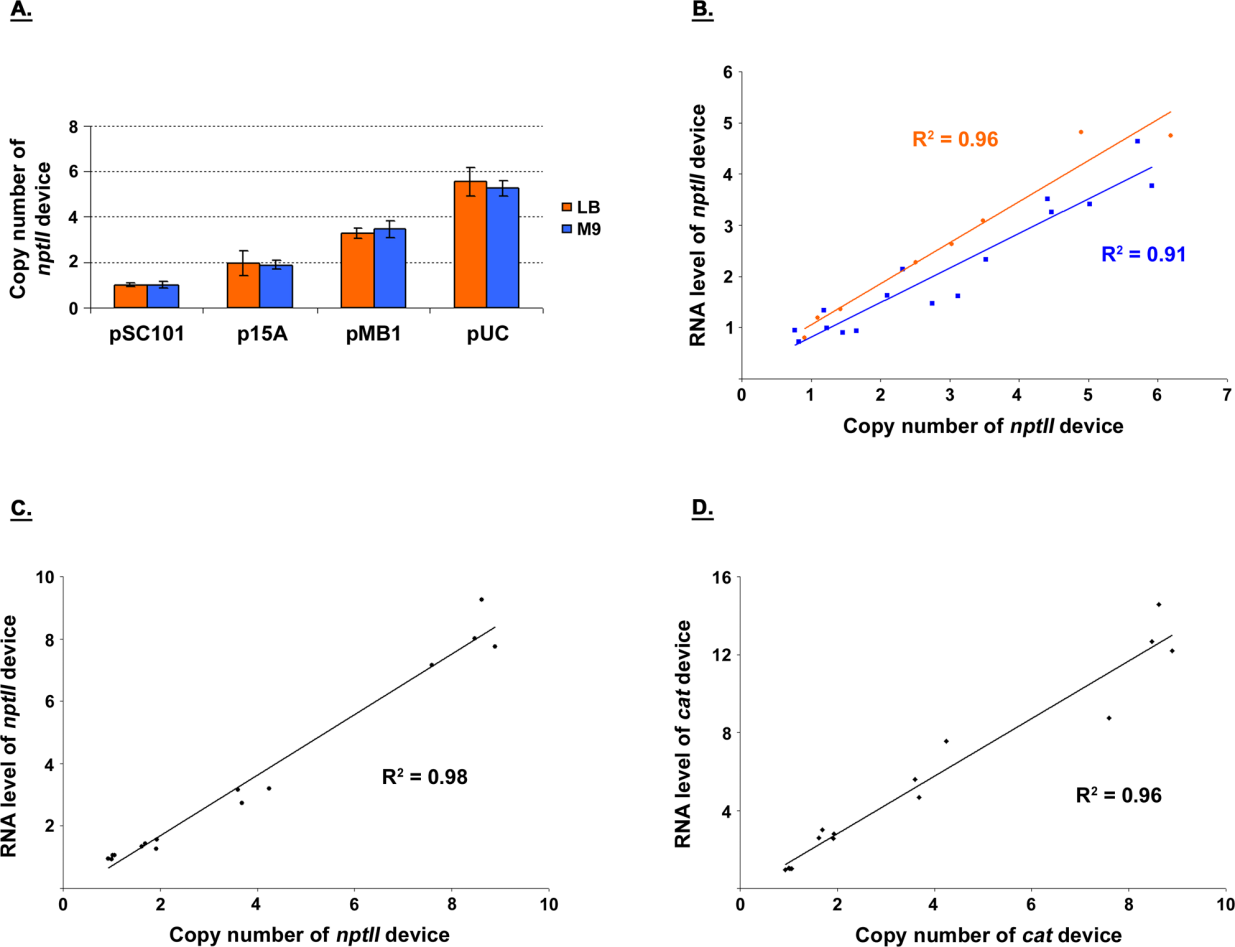
**DTC characterization of backbone and two-device containing constructs**. **A**. *nptII *copy numbers obtained from the plasmid backbone (Figure 3A) harboring the different replicons used. Each construct was tested at least in duplicate for each medium. The mean value has been reported, with the error bars denoting standard error. Values were normalized to that of the pSC101 construct, which was assigned a mean value of one. **B**. *nptII *DTC obtained from the series of plasmid backbones (Figures 3A) for LB (orange, circle) and M9 (square, blue) media. Each construct was tested at least in duplicate for each medium. **C**, **D**. *nptII *and *cat *DTCs obtained from the series of two-device plasmids (Figure 3B). The cumulative data presented were the result of two independent experiments. Constructs were tested in duplicate during each independent experiment. Three data points (and not four) are shown for the pMB1 construct because a replicate was lost during sample preparation. RNA and copy number values in panels B, C, and D were normalized to that of the pSC101 construct, with each having been assigned a mean value of one. The regression lines that minimized the sum of squared residual error are also shown, with their corresponding coefficient of determination R^2^.

The degree to which a plasmid replicates in *E. coli *is governed by its origin of replication. Using different origins, one can vary the number of copies of plasmid present in the host cell. The number of copies of the synthetic devices introduced into *E. coli *would subsequently be varied as they are harbored on the plasmid. In developing the model system for this work, the plasmid origin of replication was flanked by terminators (Figure 3A) to minimize possible transcriptional readthrough from replicons, which rely on transcription for functionality [18,19], into neighboring devices. The two terminators used in the plasmid backbone (and all of the other constructs) were the bacterial *rrnB *T1 and bacteriophage lambda t0. These strong transcriptional terminators have been widely used [20,21]. We first verified that the number of copies of a device can be varied in our system by using different replicons. To this effect, the origins of plasmids pSC101 [22], p15A [23], pMB1 [24], and pUC [24] were cloned into the backbone to determine whether the copy number of the neomycin phosphotransferase II (*nptII*) device varied (Figure 3A). This device confers resistance to the antibiotic kanamycin. The replicons from pSC101, p15A, and pMB1 are in different incompatibility groups [18,19]. As the origin of pUC is that of pMB1 with a single point-mutation [24], these two replicons are not compatible with one another. *E. coli *DH1 cells harboring plasmid backbone constructs (Figure 3A) were grown in LB and M9 minimal media as described in the Methods. The growth rate of cells was comparable among the constructs, with OD_600 nm _in the log phase doubling every ~50 and ~80 minutes in LB and M9, respectively (Table 1). With the growth rate of cells not varying with replicon (Table 1), it appears unlikely that kanamycin (which was used for selection) elicits an effect on *nptII *expression. That is, it appears that the level of *nptII *expressed at the lowest copy number of replicon pSC101 surpasses the minimum threshold necessary for cell survival. Once in mid-exponential growth, cells were harvested and total DNA extracted. Real-time quantitative PCR (qPCR) was subsequently used to determine the copy number of *nptII *for each construct [22,25-27]. Our results were similar to values reported for plasmid copy number (Figure 4A) [22-24]. It should be mentioned that the pUC origin of replication is temperature sensitive. While higher plasmid copy number values have been reported for this replicon at 37°C and 42°C, the reduced values observed here are consistent with the 30°C growth temperature used in this study [24]. Our results indicate that the copy number of a device can be varied successfully by changing the origin of replication (Figure 4A), with the range being ~6X for the constructs tested (Figure 3A). The copy number resulting from a particular origin (relative to that of pSC101) also does not appear to be impacted by the growth medium used (Figure 4A).

**Table 1:** Doubling time of cells grown in the indicated media harboring the plasmid backbone (Figure 3A)

	pSC101 replicon	p15A replicon	pMB1 replicon	pUC replicon
**LB**	45 ± 1	50 ± 2	54 ± 3	49
**M9**	80 ± 3	83 ± 3	83 ± 5	84 ± 5

Numbers indicate the time in minutes necessary for the OD_600 nm _to double in the log phase. The mean value ± 95% confidence interval as determined in duplicate has been reported.

### Linear device transfer curves obtained in *E. coli*

After verifying that the copy number of *nptII *can be varied in our system, we performed experiments to obtain its DTC. Cells that had been harvested above were used to quantify the transcript level of the *nptII *device. Total RNA was extracted from cells and transcript level quantified using real-time qPCR [28-30]. The results were plotted against the copy number values determined prior (Figure 4A), yielding the DTC (Figure 4B). RNA transcript level and copy number values have been normalized to that of the pSC101 construct, which were assigned a mean value of one in each case. The y-axis value for each data point indicates how that particular construct's steady-state transcript level compares relative to that of the pSC101 construct. Similarly, the x-axis value for each data point in the plot indicates how that particular construct's steady-state copy number compares relative to that of the pSC101 construct. Also shown in the plot are linear regressions fitted to the data. With R^2 ^> 0.9, the data suggest that the *E. coli *system response to the *nptII *device perturbation can be considered linear. The 95% confidence interval for the y-intercepts were also -0.3 - 0.8 and -0.3 - 0.5 for LB and M9 media, respectively. These included the origin, further suggesting that a linear regression was an appropriate fit for the data. That is, one cannot have *nptII *RNA transcript produced when there is no corresponding DNA present in the cell. Our results also suggest that the DTC of *nptII *can be linear under different contexts. That is, the choice of growth medium does not appear to impact system linearity (Figure 4B).

We next introduced another device into the plasmid backbone to increase the perturbation level. The goal was to see whether the presence of an additional device would lead to nonlinear DTCs. This device encoded chloramphenicol acetyl transferase (*cat*), which confers resistance to the antibiotic chloramphenicol. The *cat *device was expressed from its native promoter (Figure 3B). *E. coli *DH1 cells harboring the constructs were grown in LB medium as described in the Methods. No chloramphenicol was added to the medium during growth, with only kanamycin having been used for selection purposes. The growth rate of cells was comparable among the constructs, with OD_600 nm _doubling every ~50 minutes in the log phase (data not shown). At mid-exponential growth, cells were harvested and total RNA and DNA extracted. Relative *nptII *and *cat *transcript levels and copy number were subsequently quantified using real-time qPCR (Figures 4C and 4D). As was done for the backbone (Figure 4B), linear regressions were fitted to the data. With a R^2 ^> 0.96 for each device, the data suggest that the combined *nptII *and *cat *device perturbation level appears to be "small" enough to elicit a linear response from the *E. coli *system. The 95% confidence intervals were also -0.7 - 0.2 and -1.0 - 0.8 for *nptII *and *cat *y-intercepts, respectively, including the origin for each device once again. The fact that no chloramphenicol was present in the growth medium suggests linear transfer curve response is not due to antibiotic resistance mechanisms.

### Introduction of *gfp *genetic device led to nonlinear device transfer curves

**Figure 5:**
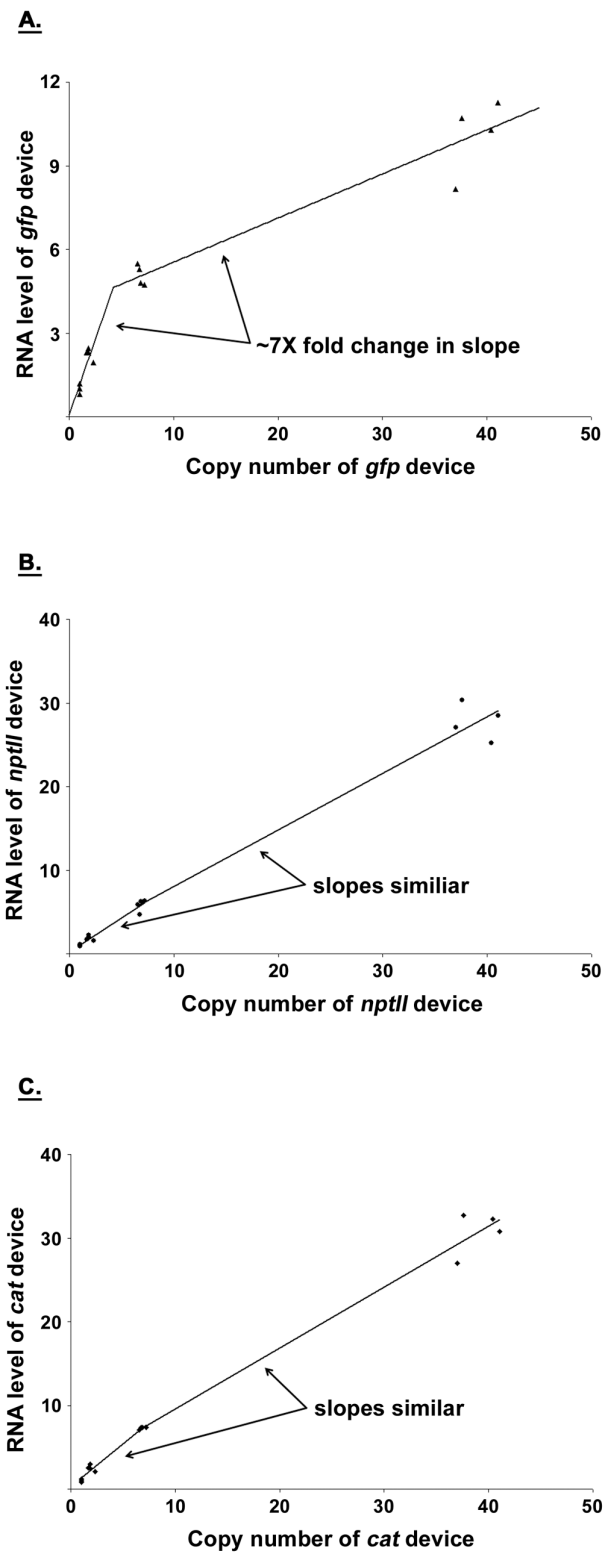
**DTC characterization of the model system**. *gfp *(**A**), *nptII *(**B**), and *cat *(**C**) DTCs obtained from the series of model system plasmids (Figure 3C). The cumulative data presented were the result of two independent experiments. Constructs were tested in duplicate during each independent experiment. Three data points (and not four) are shown for the pSC101 construct in all panels because a replicate was lost during sample preparation. RNA and copy number values were normalized to that of the pSC101 construct, with each having been assigned a mean value of one. The piecewise-linear fits that minimized the sum of squared residual error are also shown.

Considering the results described above, we constructed a model system consisting of three genetic devices to see if nonlinear DTCs would be obtained (Figure 3C). The model system is the plasmid backbone analyzed previously (Figure 3A) with two additional devices added. One genetic device encodes *cat*, while the other encodes green fluorescent protein (*gfp*). In order to investigate whether other factors besides the identity of a device's promoter impacts linear system behavior, a derivative of the constitutive bacteriophage P_L _λ promoter was used for both *cat *and *gfp *[31]. *E. coli *DH1 cells harboring the model system constructs (Figure 3C) were grown in LB medium as described in the Methods. At mid-exponential growth, cells were harvested and total RNA and DNA extracted. Relative *nptII, cat*, and *gfp *transcript levels and copy number were subsequently quantified using real-time qPCR to obtain DTCs (Figure 5). Note that the copy number of the pUC construct relative to its pSC101 counterpart was ~6X greater compared to the similar constructs in the previously studied series of plasmids (Figure 4). Unlike the latter series of plasmids, the growth rate of cells was not comparable among constructs harboring the three devices of the model system. While cells harboring the pSC101, p15A, and pMB1 constructs had doubling times comparable to one another (and similar to the ~50 minute doubling time found for the constructs used in the experiments of Figure 4, data not shown), the doubling time of cells harboring the pUC construct was ~2X greater (Table 2). It has been observed that that the plasmid copy number of constructs with ColE1-derived origins (e.g. pUC) increase under slow-growth conditions [32]. Our results are consistent with these findings.

**Table 2:** Doubling time of cells grown in LB medium harboring the different constructs in model system

	pSC101replicon	p15Areplicon	pMB1replicon	pUCreplicon
**backbone**	45 ± 1	50 ± 2	54 ± 3	49
**backbone with *cat *device**	45	51 ± 2	58 ± 4	50
**backbone with *gfp *device**	46 ± 1	53 ± 2	56 ± 1	80 ± 2
**backbone with *cat *and *gfp *devices**	46 ± 1	53	62 ± 11	103 ± 2

Numbers indicate the time in minutes necessary for the OD_600 nm _to double in the log phase. The mean value ± 95% confidence interval as determined in duplicate has been reported.

As done for the other systems, a linear regression was fitted to the *gfp *data (Figure 5A). The results suggested that the *E. coli *system response to the *gfp *device was not linear (not shown). This was due to the 95% confidence interval for the y-intercept not including the origin (i.e. 1.2 - 3). The 95% confidence interval for the y-intercept still did not include the origin if only the first three constructs (i.e. the ones with the pSC101, p15A, and pMB1 replicons) were considered (data not shown). This suggested a piecewise-linear model for the data, with the first segment consisting of data points for the pSC101, p15A constructs (where the 95% interval for the y-intercept included the origin, data not shown) and the second segment data points for the pMB1, pUC constructs. The piecewise-linear approximation is used in electrical engineering to model nonlinear transfer curves [33]. To arrive at a piecewise-linear model in a systematic manner, the problem was approached as a nonlinear least squares optimization [34]. The NLIN Gauss-Newton procedure in SAS was used to fit the data to a piecewise-linear model consisting of two segments with unknown slopes and an unknown breakpoint (Figure 5A). The algorithm was not forced to go through the origin. This way, the appropriateness of the fit could later be verified by noting the y-intercept obtained from the slopes and breakpoint numerically computed by the NLIN procedure. No noticeable normality or variance issues were observed after analyzing the residuals in SAS (data not shown), strengthening the argument for the appropriateness of the model. A y-intercept of 0.07 was obtained, which is approximately equal to the origin. The change in slope between the two segments (~7X fold) was taken as a means to report the nonlinearity observed in the *gfp *DTC.

As was done for *gfp*, SAS was used to fit piecewise-linear models to the data for the *nptII *and *cat *devices (Figure 5B and 5C, respectively). Unlike the former, however, the fits that minimized the sum of squared residuals had the first segment consisting of data points for the pSC101, p15A, and pMB1 constructs (data not shown). The second segment could, thus, not be determined because the pUC construct remained as the only available point (i.e. one needs two points to fit a line). As an approximate solution to this problem, a piecewise-linear model was determined for each device by fitting a linear regression to the pSC101, p15A, pMB1 and pMB1, pUC constructs for the first and second segments, respectively (Figures 5B and 5C). The two segments for the *nptII *and *cat *devices had similar slopes. This was noticeably smaller than the ~7X fold change observed for *gfp *(Figure 5A).

### Superposition lost at higher expression levels with the addition of *gfp*

**Figure 6:**
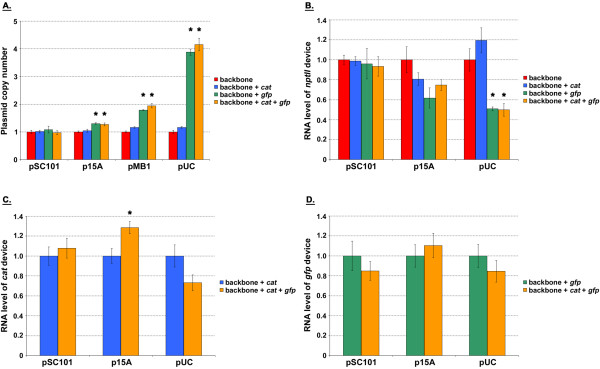
**Testing for the presence of superposition in the model system**. For each of the indicated replicons, plasmid copy number (**A**) and *nptII *transcript level per plasmid copy (**B**) were determined for empty backbone and backbone harboring *cat *or/and *gfp *devices. Values for each replicon were normalized to that of the empty backbone construct, which was assigned a mean value of one. The difference in value between empty and device harboring backbone was determined statistically for each replicon (P < 0.05, t-test). Asterisks indicate significant difference. **C**. For each of the indicated replicons, *cat *transcript level per plasmid copy was determined for backbone containing either *cat *device alone or both *cat *and *gfp *devices. Values for each replicon were normalized to that of the backbone construct with only *cat *device present, which was assigned a mean value of one. The difference in value between backbone containing either *cat *device alone or both *cat *and *gfp *devices was determined statistically for each replicon (P < 0.05, t-test). Asterisks indicate significant difference. **D**. For each of the indicated replicons, *gfp *transcript level per plasmid copy was determined for backbone containing either *gfp *device alone or both *cat *and *gfp *devices. Values for each replicon were normalized to that of the backbone construct with only *gfp *device present, which was assigned a mean value of one. The difference in value between backbone containing either *gfp *device alone or both *cat *and *gfp *devices was determined statistically for each replicon (P < 0.05, t-test). No significant differences were observed. For all panels, the mean value as determined from two independent experiments (constructs were tested in duplicate during each independent experiment) has been reported. Error bars denote standard error.

We next performed superposition experiments to verify the DTC results of the previous section and to determine whether the *E. coli *system can indeed behave as a linear system under "small" perturbation conditions. If the nonlinear *E. coli *system can be approximated as a linear one, the perturbing devices may be studied independent of one another (Figure 2C). This, in turn, would allow one to predict the response of *E. coli *to the complete system (backbone with both *cat *and *gfp *devices) from characterization data of the individual devices. In other words, the addition of new devices would not impact the expression levels of the devices present prior. *E. coli *DH1 cells harboring either the empty plasmid backbones or the various backbones containing *cat *or/and *gfp *devices were grown in LB medium. At mid-exponential growth, cells were harvested and total DNA extracted. Plasmid copy number was subsequently quantified using real-time qPCR (Figure 6A). Our results indicated that the plasmid copy number was unaffected by the addition of *cat *and/or *gfp *devices to the backbone with a pSC101 origin. Device addition, however, began to have an impact at higher copy numbers. The change in plasmid copy number was most pronounced by the addition of the *gfp *device, with those resulting from *cat *not being statistically significant even with the pUC replicon. While the growth rate data (Table 2) also support this finding, the numbers suggest that a change in the plasmid copy is not necessarily reflected by a corresponding change in the cell doubling time.

The larger nonlinearity observed in the *gfp *DTC (Figure 5A) interestingly also manifested itself in superposition experiments involving device transcript levels. Cells containing the pSC101, p15A, and pUC origins that had been harvested above were used to quantify the transcript levels of the different devices. The latter two replicons were chosen so as to have data points on either side of the DTC breakpoints (Figure 5). The pSC101 origin was selected to investigate whether superposition observed at the plasmid copy level (Figure 6A) also applied to device transcript levels. Total RNA was extracted from cells and transcript levels quantified using real-time qPCR (Figures 6B, 6C, and 6D). As the plasmid copy varied among the constructs for a particular replicon (Figure 6A), transcript levels were not only normalized to the endogenous 16S but also to the plasmid copy number. That is, values reported are RNA produced per unit plasmid. Superposition would be in effect if the amount of RNA produced by the *nptII *device (Figure 6B) did not change after additional devices had been introduced into the plasmid. That is with superposition, if the plasmid backbone harboring genetic device *nptII *led to the production of a certain amount of that device's RNA, one would obtain the same amount upon addition of *cat *and/or *gfp *devices. Similar arguments apply to superposition for the *cat *and *gfp *devices (Figures 6C and 6D, respectively). As was the case for plasmid copy (Figure 6A), our results indicated that *nptII *transcript level is unaffected by the addition of *cat *and/or *gfp *genetic devices to a plasmid with a pSC101 replicon (Figure 6B). Device addition began to have an impact at higher copy numbers. The changes in *nptII *transcript level were, once again, the most pronounced by the addition of the *gfp *device, with those resulting from *cat *not being statistically significant even with the pUC origin. The data for *cat *and *gfp *RNA exhibited a similar pattern. Once again, *cat *or *gfp *transcript level was unaffected by the addition of the other device to a plasmid with a pSC101 replicon (Figures 6C and 6D). The addition of *gfp*, however, affected *cat *RNA at the higher copy numbers (Figure 6C). This was not the case in the reverse direction. That is, *cat *device addition did not impact *gfp *RNA at the higher copy numbers of p15A and pUC (Figure 6D). These results suggest that the extent of the changes brought about by *gfp *is large enough to mask those caused by the addition of *cat*.

## Discussion

Our results indicate that the *E. coli *biological system can exhibit linear system behavior (Figure 6). In the model system presented in this work, the necessary condition with all three genetic devices present was to use a plasmid backbone harboring the pSC101 replicon. That is, our experimental results showed superposition to be present at this copy number. The presence of superposition, however, was not only a consequence of having used the pSC101 origin. In the absence of the *gfp *device, superposition was found even with a pUC origin (Figure 6). The finding that superposition is possible under different contexts is important. It suggests that the nonlinearity in the *E. coli *system is not complex to the point of preventing design efforts to elicit a linear system response.

A simple mathematical model that captures our DTC results can be derived by noting the rate of change of a molecular entity is a synthesis term minus a degradation term. Assuming that the synthesis of RNA is proportional to the amount of DNA present and that its degradation is proportional to the amount of RNA, the equation for RNA becomes [35](1)

where α and β are proportionality constants that capture the synthesis and degradation rates, respectively. Under steady-state conditions, the left hand side of equation (1) becomes zero and one arrives at the following equation(2)

where *RNA_SS _*and *DNA_SS _*are the steady-state RNA and copy number of the encoding DNA, respectively. The nonlinearity observed in the piecewise-linear DTCs (Figure 5) may, thus, be modeled by a change in the  slope term of equation (2). That is, the synthesis and/or degradation rate varies for the devices at higher copy numbers. Analysis of RNA degradation after cells had been treated with rifampicin did not reveal a noticeable change in the decay rates of *gfp *and *cat *transcripts at the higher copy number of pUC relative to p15A (data not shown). This suggests that the larger nonlinearity observed in *gfp *is due to a modulation in the synthesis rate. The fact that *gfp *and *cat *have identical P_L _promoters in our model system further suggests that the mechanism involved does not affect the initiation of transcription. Perhaps, the stringent response is implicated in this matter. Previous work has indicated that the *E. coli *stringent response can differentially impact the elongation rate of transcripts [36]. We were unable to grow cells harboring the model system plasmids (Figure 3C) with the pMB1 and pUC replicons in M9 minimal medium (data not shown), suggesting that cells might be in a starvation like condition at the higher copy numbers. This is also supported by our growth rate data in LB medium, with the doubling time increasing at the higher copy numbers (Table 2). Gene sequence has been shown to influence transcriptional pausing of RNA polymerase in the presence of guanosine tetraphosphate (ppGpp) [37]. Perhaps, the coupling present between transcription and translation in *E. coli *facilitates this effect, with ribosomal ppGpp synthesis affecting upstream RNA polymerase that is in the process of transcript elongation. Cooperative activity between RNA polymerase and ribosomes has been shown to modulate the elongation rate of transcripts in *E. coli *[38], with this linkage involving the NusE-NusG complex [39]. It also needs to be acknowledged that copy number was the only perturbing factor considered in our study. The *cat *and *gfp *genes were expressed as done previously [31] (see below). As such, the devices have different RBS sequences/strengths. Promoter strength, RBS strength, and codon usage may be coupled perturbing factors because of the cooperative activity between RNA polymerase and ribosomes. Indeed, a range of promoters, RBS strengths, and codon usage need to be used to better elucidate the mechanism underlying the large observed nonlinearity in *gfp*'s DTC (Figure 5).

Our results do, however, suggest that the transfer curve-based framework has application in the engineering of biological systems. We observed a correspondence between DTC nonlinearity and the break down of linear system behavior. That is, the *gfp *device was found to elicit a more nonlinear DTC response from the *E. coli *system than the other tested devices (~7X change in slope as compared to no change for *nptII *and *cat *devices, Figure 5), which was reflected in superposition being lost when *gfp *was present at copy numbers above the pSC101 level. While a change in growth rate offers an alternate gauge for nonlinearity, it does not appear to provide one with the same level of accuracy. The doubling time as monitored by OD_600 nm _only began to change noticeably with the pUC replicon (Table 2), failing to indicate changes to copy number (Figure 6A) and transcript (Figures 6B, C, and 6D) due to *gfp *at the other origins. This indicates the significance of quantitative techniques (such as DTCs) to synthetic biology characterization efforts because growth rate alone is unable to accurately capture changes that take place due to device addition. DTCs may have application in the general characterization of devices. A device could be characterized by cloning it into a standard plasmid and its copy number varied by way of different replicons. Based on the nonlinearity gauged from its resulting DTC, one may subsequently be able to determine whether the device is well suited for eliciting a predictable, linear response from *E. coli *when used in combination with other devices.

Determining factors that impact linear system behavior in *E. coli *would also be of benefit to synthetic biology. Such knowledge may enable the construction of biological systems using superposition because guidelines for the conditions necessary that ensure linear system behavior would be available. In this study, we focused on device copy number as the perturbing factor. Promoter strength is another important factor (as are RBS strength, gene length, codon usage, and product function). A library of constitutive promoters has been characterized using the *cat *and *gfp *genes [31]. By expressing *cat *and *gfp *in the manner done in that study, the model system constructed in this work can be used to investigate the effect promoter strength has on linear system behavior. Our results appear to suggest that the identity of a device's promoter is not the only factor that impacts linearity in its RNA expression profile. While *gfp *had a promoter identical to that of *cat*, the former was the most nonlinear in our three-device model system (Figure 5). In fact, *cat *and *nptII *had similar DTCs in our three-device model system, but yet had different promoters. Comparison of the *cat *DTCs in our two-device (Figure 4D) and three-device (Figure 5C) constructs also supports this premise. The *cat *DTC was primarily linear for both cases. The *cat *device in one experiment, however, had its native promoter (Figure 4D), while the P_L _promoter was used in the other (Figure 5C). Comparison of the DTC results of our two-device (Figures 4C and 4D) and three-device (Figures 5B and 5C) constructs also suggest that the amount of nonlinearity in the *E. coli *system response to devices harboring *nptII *and *cat *genes is not impacted greatly by slow-growth conditions. Our results from the backbone, two-device, and three-device constructs also suggest that the DTC slope (which is the transfer curve gain) may act as a useful metric for characterizing promoter strength of a gene. The gain of the native *nptII *promoter was found to be ~1 in the various constructs tested (Figures 4B, 4C, and 5B), with similar values having been found irrespective of the choice of growth medium (Figures 4B) or a change in growth rate (Figures 4C and 5B).

Small-signal linearization techniques may also have application to other aspects of biological system behavior. Input-output relationships can be defined and experimentally measured to generate transfer curves, where piecewise linear models may subsequently be employed to determine the linear range of the system. Examples could include inducer concentration to activated transcription factor, activated transcription factor to RNA, and RNA to protein transfer curves. Measuring input-output characteristics and applying small-signal linearization techniques have the potential of reducing the complex mathematical equations used to model biological interactions to their simplest form; thereby, permitting predictable, quantitative behavior predictions. The limitation of small-signal linearization techniques is that the linearity property needs to be checked. As was observed in our model system, however, some systems can have a linear regime. So long as experiments are performed within this regime, one can avoid nonlinear effects and apply the simplifications associated with a small-signal linear model. And even if the system is to be operated in the nonlinear regime, it may be possible to introduce nonlinear correction factors to the obtained small-signal linear model. In Equation (2), for instance, this can be modeled by having the magnitudes of α and/or β dependent on the copy number (as opposed to constant values). With our results suggesting that the transfer curve and small-signal concepts used in electrical engineering can likewise be employed towards biological systems, the application of other concepts may also be of benefit to synthetic biology. The transfer curve concept is primarily of use in studying the steady-state behavior of a system. Linear systems can also be studied in the frequency domain by using Fourier techniques, which enable engineers to predict time-domain system response. These techniques have been used previously to study the yeast osmo-adaptation system [40]. Indeed, the application of analysis and design techniques of other established engineering disciplines may enable the systematic forward engineering of biological systems for improved biotechnology applications.

## Conclusions

We have presented a model system and framework to investigate linear system behavior in *E. coli*. With all three genetic devices present in the model system, we show the existence of superposition at the pSC101 copy number level. In the absence of the *gfp *device, linear system behavior was present even with a pUC replicon. The amount of nonlinearity in our model system appears to be biased towards the *gfp *device. This is in spite of the fact that the *gfp *and *cat *devices have identical constitutive promoters. Such a finding suggests additional factors besides promoter strength impact the amount of nonlinearity in a device's steady-state RNA expression profile. Our developed DTC method may have application in the systematic testing of device nonlinearity to determine whether a device will give a predictable output when used in combination with other devices. This, in turn, may enable the design and construction of biological systems with predictable, quantitative behavior from smaller elements characterized in isolation.

## Methods

### Bacterial strains, media, and enzymes

*E. coli *DH10B and DH5α were used for cloning. *E. coli *DH1 was used for expression work. Luria-Bertani (LB) media was made as described in [41]. M9 minimal media + 0.5% glucose supplemented with micronutrients was made as described in [42]. Restriction enzymes and T4 DNA ligase were purchased from New England Biolabs, with digestion and ligation reactions performed as recommended by the enzyme manufacturer. PCR reactions were performed with Phusion polymerase from Finnzymes, and the primers used were synthesized by Integrated DNA Technologies, Inc. The composition of the PCR reactions, cycle times, and temperatures followed those suggested by the enzyme manufacturer. PCR products were sequenced once cloned into the respective plasmids to ensure that no mutations had been introduced during the amplification process. In cases where single digest cloning was performed, sequencing was also used to select for constructs with inserts in the desired orientation.

### Plasmid construction

The plasmid backbones with the replicons of pSC101, p15A, pMB1, and pUC (Figure 3A) were constructed using standard molecular biology techniques [41]. These plasmids were named pAmin81 [GenBank:HQ283398], pAmin78 [GenBank:HQ283399], pAmin79 [GenBank:HQ283400], and pAmin80 [GenBank:HQ283401], respectively.

The construction of the two-device series of plasmids (Figure 3B) proceeded as follows. PCR was used to obtain a spacer and the *cat *gene (with its corresponding native promoter) as inserts. Primers lacZ_1_F and lacZ_1_R (Table 3) were used to obtain the spacer, with p50 gl [43] having been used as template. A spacer was used to create spatial separation between the neighboring *nptII *and *cat *devices, and to not have the devices right next to each other. The spacer sequence consisted of a ~600bp fragment taken from within the coding region of the bacterial *lacZ *gene. The *cat *gene was obtained by using the primers cat_wt_F and cat_wt_R (Table 3), with pACYC184 having served as template. The spacer and *cat *inserts were digested with *AvrII, XbaI *and *XbaI, SacI*, respectively, and ligated into a *AvrII, SacI *digested pAmin81 in a three-fragment ligation reaction. The *cat *genetic device (complete with spacer and terminator) was subsequently transferred into pAmin78, pAmin79, pAmin80, and pAmin81 using *MluI *single digest to arrive at the desired series of plasmids (Figure 3B).

**Table 3:** List of PCR primers used in the cloning of the plasmids constructed in this study

Primer	Sequence
lacZ_1_F	tattatctcgagtacctaggggtaacagtttctttatgg
lacZ_1_R	tattattctagattcgctggtcacttcgatggtttg
cat_wt_F	tattattctagagacgtcgaataaatacctgtgacggaag
cat_wt_R	tattaagagctcaggcctaataactgccttaaaaaaattacg
cat_orf_F	tattatggtacctttcaggagctaaggaagctaaaatg
cat_orf_R	taataaacgcgtccaataactgccttaaaaaaattacg
PL_F	tattatgacgtctccctatcagtgatagagattgacatc
lacZ_2_F	tattaacctaggaggatccatgttgccactcgc
lacZ_2_R	taataagacgtcatcggtcagacgattcattg
gfp_F	tattatggtaccgcatgcgtaaaggagaagaacttttcactggagttgtcc
gfp_R	taataaaagcttattaaactgatgcagcgtagttttcgtcgtttgctgcaggccttttg
gfp_2_R	tattaagagctcgaagtgcttcaagcttattaaactgatgcagcgtag

The construction of the model system series of plasmids (Figure 3C) proceeded by first creating a series of *cat *device (with the P_L _promoter) containing plasmids. PCR was used to obtain the *cat *open-reading frame as insert. The primer pairs used were cat_orf_F and cat_orf_R (Table 3), with pACYC184 having served as template. This insert was digested with *KpnI, MluI*, and ligated into pZE21 [44] to create pAmin92. PCR was subsequently used to obtain the *cat *gene (with the P_L _promoter) as insert using the primer pairs PL_F and cat_wt_R (Table 3) and pAmin92 as template. This insert was digested with *AatII, StuI*, and ligated into the two-device (Figure 3B) series of plasmids to yield a series of *cat *device (with the P_L _promoter) containing constructs. Next, work began on constructing a *gfp *device (with the P_L _promoter) containing construct with pSC101 as the origin. This plasmid was called pAmin81+*gfp*_PL_. PCR was used to obtain a spacer (different in sequence from that above) and the *cat *gene as inserts. Primer pairs lacZ_2_F and lacZ_2_R (Table 3) were used for the spacer, and p50 gl served as the template. The *cat *gene was obtained by using the primers cat_wt_F and cat_wt_R (Table 3), with pACYC184 having served as template. The spacer here was to create spatial separation between the neighboring *cat *and *gfp *devices, and consisted of a ~600 bp fragment taken from within the coding region of the bacterial *lacZ *gene. The spacer and *cat *inserts were digested with *AvrII, AatII *and *AatII, SacI*, respectively, and ligated into a *AvrII, SacI *digested pAmin81 in a three-fragment ligation reaction to create pAmin93. The *cat *genetic device (complete with spacer and terminator) was subsequently transferred into pAmin81 to create pAmin99. PCR was next used to obtain the *gfp *open-reading frame as insert. Primer pairs gfp_F and gfp_R (Table 3) were used, with BBa_E0044 [5] serving as the template. This insert was digested with *KpnI, HindIII*, and ligated into pZE21 to create pAmin100. PCR was then used to obtain the *gfp *gene (with P_L _promoter) using the primer pairs PL_F and gfp_2_R (Table 3) and pAmin100 as template. The creation of pAmin81+*gfp*_PL _subsequently proceeded by performing a three-fragment ligation reaction of this fragment digested with *AatII, SacI*, the ~2.5 kb fragment released from a *AvrII, SacI *digested pAmin93, and the ~2 kb fragment released from a *AvrII, AatII *digested pAmin99. The *gfp *genetic device (complete with spacer and terminator) was finally transferred from pAmin81+*gfp*_PL _into the *cat *device (with the P_L _promoter) containing series of plasmids described prior using *BamHI*, creating the desired series of plasmids (Figure 3C). Sub-cloning was used in order to arrive at the *gfp *device (with the P_L _promoter) containing constructs with the other three origins of replication. More specifically, the origins released from a *AvrII, SmaI *digested pAmin78, pAmin79, and pAmin80 were ligated into pAmin81+*gfp*_PL _to yield pAmin78+*gfp*_PL_, pAmin79+*gfp*_PL_, and pAmin80+*gfp*_PL_.

### Bacterial growth conditions

*E. coli *DH1 cells were grown overnight at 30°C, 200 rpm shaking after inoculating 5 mL cultures of LB media (supplemented with 50 μg/mL kanamycin) with single colonies from freshly streaked plates. After sub-culturing (1:50) into shake flasks containing 50 mL of either M9 minimal or LB media (supplemented with 50 μg/mL kanamycin), cells were grown at 30°C, 200 rpm shaking until an OD_600 nm _of 0.3-0.4 was reached to approximate steady-state conditions. At this time, 1 mL of cells were added to ice chilled tubes with 100 μL of 10% phenol:90% EtOH stop solution [45], mixed, spun down, supernatant removed, and total RNA isolation proceeded immediately thereafter. Another 1 mL of cells were spun down, supernatant removed, and cell pellets subsequently frozen for total DNA isolation at a future date.

### Bacterial total RNA isolation to quantify *nptII, cat*, and *gfp *expression levels

Bacterial cell pellets were resuspended in 700 μL buffer RLT (Qiagen), to which beta-mercaptoethanol had been added according to the manufacturer's instructions. Cells were subsequently lysed using 0.1 mm diameter glass beads in the Mini-Beadbeater-8 (Biospec). Following lysis, tube contents were spun down and 500 μL of lysate was transferred to new tubes. Total RNA extraction then proceeded by adding 500 μL of 25:24:1 phenol:chloroform:isoamyl alcohol, vortexing vigorously for ~1 min, allowing the tubes to sit at bench for a few minutes subsequent, and centrifugation for 15 min at 12000 × g, 4°C. Next, 300 μL of the upper aqueous phase was transferred to a new tube containing 300 μL nuclease free water. RNA extraction continued by adding 600 μL of chloroform to each tube, vigorous vortexing for ~1 min, allowing the tubes to sit at bench for a few minutes subsequent, and centrifugation for 15 min at 12000 × g, 4°C. Next, 300 μL of the upper aqueous phase was transferred to a new tube. Following chloroform extraction, total RNA was ethanol precipitated overnight, washed with 70% ethanol, and finally resuspended in 30 μL of nuclease free water. RNA concentration and purity were assayed using a Nanodrop spectrophotometer, and integrity examined on 2% agarose gels.

### cDNA synthesis and real-time qPCR quantification of cellular *nptII, cat*, and *gfp *transcript levels

Total RNA extracted was treated with Turbo DNase (Ambion) to reduce DNA contamination. First-strand cDNA was synthesized by using reverse gene-specific primers (Table 4) and SuperScript III Reverse Transcriptase (Invitrogen) following the manufacturer's instructions. Transcript levels were normalized to that of endogenous 16S rRNA. The primer sets specific to *nptII, cat, gfp*, and 16S rRNA (Table 4) amplified a single product of the expected size as confirmed by the melting temperatures of the amplicons. Real-time qPCR was conducted on a BioRad iCycler with 96-well reaction blocks in the presence of SYBR Green under the following conditions: 1X iQ SYBR Green Supermix (BioRad), 150 nM *nptII*, 300 nM *cat*, 100 nM *gfp*, or 500 nM 16S primers in a 25 μL reaction. Real-time qPCR cycling was 95°C for 3 min, followed by 40 cycles of 30 sec at 95°C, 30 sec at 60°C, and 30 sec at 72°C. Threshold cycles (Ct) were determined with iCycler (BioRad) software for all samples. A standard curve was prepared for quantification. For this purpose, a fourfold dilution series of a total of seven dilutions was prepared from a digested total DNA sample, and each dilution was subjected to qPCR analysis in triplicate with either the *nptII*-, *cat*-, *gfp*-, or 16S-specific primers. Obtained Ct values were used by the iCycler software package to plot a standard curve that allowed quantification of *nptII, cat, gfp*, or 16S in the total RNA samples (i.e. unknowns) relative to the RNA sample used to prepare the standard curve.

**Table 4:** List of real-time qPCR primers used in this study

Primer	Sequence	Reference
qpcr_nptII_F	gcgttggctacccgtgatat	[48]
qpcr_nptII_R	aggaagcggtcagcccat	[48]
qpcr_cat_F	cgcaaggcgacaaggtg	[49]
qpcr_cat_R	ccatcacaaacggcatgatg	[49]
qpcr_gfp_F	aagcgttcaactagcagacc	[30]
qpcr_gfp_R	aaagggcagattgtgtggac	[30]
qpcr_16S_F	ccggattggagtctgcaact	[24]
qpcr_16S_R	gtggcattctgatccacgattac	[24]

### Bacterial total DNA isolation to quantify plasmid copy number

The DNA isolation method reported in the previous publications [24,46] was adopted. Bacterial cell pellets were resuspended in 400 μL of 50 mM Tris/50 mM EDTA, pH 8, by vortex. Cell membranes were permealized by the addition of 8 μL of 50 mg/mL lysozyme (Sigma) in 10 mM Tris/1 mM EDTA, pH 8, followed by incubation at 37°C for 30 min. To complete cell lysis, 4 μL of 10% SDS and 8 μL of 20 mg/mL Proteinase K solution (Invitrogen) were added to each tube, mixed with a syringe with 21 gauge 1.5 inch needle, and incubated at 50°C for 30 min. Proteinase K was subsequently heat inactivated at 75°C for 10 min, and RNA was digested with the addition of 2 μL of 100 mg/mL RNase A solution (Qiagen) followed by incubation at 37°C for 30 min. Total DNA extraction then proceeded by adding 425 μL of 25:24:1 phenol:chloroform:isoamyl alcohol, vortexing vigorously for ~1 min, allowing the tubes to sit at bench for a few minutes subsequent, and centrifugation for 5 min at 12000 × g, 4°C. Next, 300 μL of the upper aqueous phase was transferred to a new tube using a wide-opening pipet tip. DNA extraction continued by adding 400 μL of chloroform to each tube, vigorous vortexing for ~1 min, allowing the tubes to sit at bench for a few minutes subsequent, and centrifugation for 5 min at 12000 × g, 4°C. Next, 200 μL of the upper aqueous phase was transferred to a new tube using a wide-opening pipet tip. Following chloroform extraction, total DNA was ethanol precipitated overnight, washed with 70% ethanol, and finally resuspended in 40 μL of nuclease free water. DNA concentration and purity were assayed using a Nanodrop spectrophotometer, and integrity examined on 1% agarose gels.

### Real-time qPCR quantification of plasmid copy number

Primer sets specific to the *nptII *and 16S rDNA genes were used (Table 4). These primers amplified a single product of the expected size as confirmed by the melting temperatures of the amplicons. The *nptII *gene is a single-copy gene of the plasmids characterized in this study, while 16S is a multi-copy gene of *E. coli *chromosomal DNA [47] and was used for normalization purposes [24,26]. Total DNA isolated from each strain was first digested overnight using EcoRI (New England Biolabs) at 37°C. Real-time qPCR was conducted on a BioRad iCycler with 96-well reaction blocks in the presence of SYBR Green under the following conditions: 1X iQ SYBR Green Supermix (BioRad), 150 nM *nptII*, or 500 nM 16S primers in a 25 μL reaction. Real-time qPCR cycling was 95°C for 3 min, followed by 40 cycles of 30 sec at 95°C, 30 sec at 60°C, and 30 sec at 72°C. Threshold cycles (Ct) were determined with iCycler (BioRad) software for all samples. A standard curve was prepared for quantification. For this purpose, a fourfold dilution series of a total of seven dilutions was prepared from a digested total DNA sample, and each dilution was subjected to qPCR analysis in triplicate with either the *nptII*- or 16S-specific primers. Obtained Ct values were used by the iCycler software package to plot a standard curve that allowed quantification of *nptII *or 16S in the digested total DNA samples (i.e. unknowns) relative to the DNA sample used to prepare the standard curve.

## List of abbreviations used

*cat*: chloramphenicol acetyl transferase; DTC: device transfer curve; *gfp*: green fluorescent protein; ppGpp: guanosine tetraphosphate; *nptII*: neomycin phosphotransferase II; qPCR: quantitative PCR; RBS: ribosome binding site.

## Competing interests

The authors declare that they have no competing interests with respect to this work. JDK has a financial interest in Amyris and LS9.

## Authors' contributions

MH conceived the study, designed and performed all experiments, analyzed the data, and wrote the manuscript. PRG contributed in discussing the study, analysis of the experimental results, and edited the manuscript. JDK contributed in the design and coordination of the study, analysis of the experimental results, and edited the manuscript. All authors read and approved the final manuscript.
